# Anti-pyocyanin Antibody Exhibits Cytotoxicity Protective Effects on Macrophages: A Promising Innovative Therapeutic Approach for *Pseudomonas aeruginosa* Infections

**DOI:** 10.1021/acsptsci.5c00187

**Published:** 2025-11-06

**Authors:** Bárbara Rodríguez-Urretavizcaya, Tamás Posvai, Lluïsa Vilaplana, María-Pilar Marco

**Affiliations:** † Nanobiotechnology for Diagnostics (Nb4D), Institute of Advanced Chemistry of Catalonia, IQAC−CSIC, Jordi Girona, 18-26, 08034 Barcelona, Spain; ‡ CIBER de Bioingeniería, Biomateriales y Nanomedicina (CIBER-BBN), Av. Monforte de Lemos, 3-5. Pabellón 11, Planta 0, 28029 Madrid, Spain

**Keywords:** *Pseudomonas aeruginosa*, pyocyanin, monoclonal antibody, therapy, cell viability, cytokines

## Abstract

*Pseudomonas aeruginosa* is considered one of the most threatening pathogens worldwide, due to its high adaptability, which leads to resistance to classical antimicrobials. This fact has driven the development of new therapeutic strategies to reduce multiresistant strains and to minimize infection progression. In this context, the protective effect of a monoclonal antibody (mAb122) specific to pyocyanin (PYO), a key virulence factor of *P. aeruginosa*, was studied *in vitro*. Quenching PYO may reduce *P. aeruginosa* pathogenesis and partially lessen host immune dysregulation by impairing cytokine production. With this aim, murine macrophages were challenged with different PYO concentrations to assess their cytotoxicity by evaluating different cell viability hallmarks. Subsequently, the protective effect of mAb122 was studied on the PYO-treated cells. The addition of mAb122 significantly increased the percentage of viable cells compared to those treated just with the virulence factor (4.34- to 11.07-fold increase in MH-S and RAW 264.7 cells, respectively). Moreover, the PYO immunomodulatory effect and the outcome of mAb122 addition on the host response were also studied by measuring relevant cytokines in cell media. Results showed that mAb122 treatment, rather than reversing PYO impairment in cytokine production, either maintained the levels or triggered an increase, depending on the specific cytokine examined. Thus, the significant rise in cell viability and the nontoxic effect of mAb122 itself *in vitro* place PYO mAb as a promising candidate for *in vivo* testing as a potential therapeutic agent. However, its effects on the host immune system should be carefully studied and minimized.

## Graphical abstract

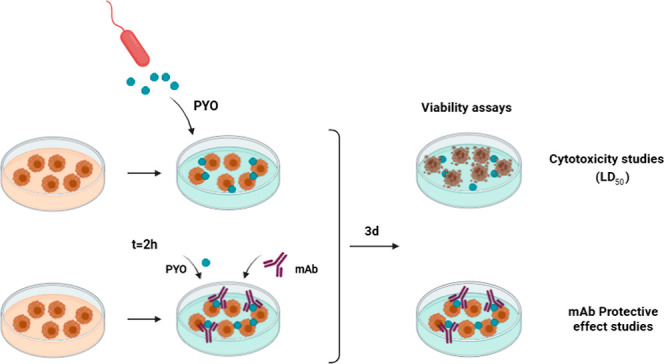

## 1 Introduction

According to the World Health Organization (WHO), infectious diseases are a leading cause of death worldwide. Thus, in 2024, three types of infectious diseases, COVID, lower tract respiratory infections (LTRIs), and tuberculosis, were ranked in the top ten causes of death list. More precisely, LTRIs have been designated as the fifth leading cause of death globally.^1^ In fact, chronic obstructive pulmonary disease (COPD) and pneumonia were responsible in 2021 for approximately 324,000 deaths in Europe, remaining the most frequent causes of death from communicable diseases.^2^ Moreover, the prevalence and mortality caused by these infections is likely to rise in the coming years as a result of the increase in antimicrobial resistance (AMR).^3^ AMR processes were described more than 70 years ago after the appearance of sulfonamides in 1937.^4,5^ This is primarily due to the inadequate use of broad-spectrum antibiotics and the prolonged overuse of these treatments.^6–8^ Furthermore, in the past decade, only a few new antibiotics have entered the market because of the complexity and high costs associated with drug development.^4,5,9,10^ Therefore, multiresistant microorganisms have emerged as a worldwide public health concern, causing substantial economic losses.^6^

In this context, nosocomial infections, also referred to as hospital-acquired/associated infections (HAIs), have also increased in number.^11^ These infections are mainly caused by multidrug-resistant pathogens included in the WHO Bacterial Priority Pathogens List (BPPL)^12^ such as *Escherichia coli*, *Staphylococcus aureus*, *Enterococcus* species, *Pseudomonas aeruginosa*, and *Klebsiella* species being the most commonly isolated ones. Nosocomial infections account worldwide for approximately 10% of all hospitalized patients. From this group, the Gram-negative bacterium *P. aeruginosa* contributes to 9% of all HAI infections.^11^ Due to its high adaptability to diverse natural environments and its metabolic versatility, *P. aeruginosa* has evolved resistance to most of the available antibiotics resulting in 2700 estimated deaths in the US (year 2017)^13^ and 3210 in Europe (year 2020).^14^

It has been reported that *P. aeruginosa* utilizes intrinsic (membrane permeability, efflux pumps) and adaptive resistance mechanisms to counteract antibiotic effects.^15^ In this bacterial species, the adaptive resistance mechanism is mainly based on the formation of a dense bacterial community attached to a solid surface named biofilm (mucoid phenotype).^16–18^ When *P. aeruginosa* adopts this phenotype, it is able to induce chronic infections. These chronic infections are characterized by the overproduction of exopolysaccharides and the reduction of motility and virulence factor levels.^19,20^ All these phenotypic changes induce AMR^21,22^ and increase persister cell generation.^23–25^ However, despite the rise of multidrug-resistant strains and the ineffectiveness of the current antimicrobials, most therapeutic strategies used for treating any suspected case of *P. aeruginosa* are still based on the administration of these types of drugs, especially β-lactams alone or in combination with compounds from other antibiotic families.^15,26–28^ Thus, all of these facts point to the need of developing novel and alternative effective therapies against *P. aeruginosa* infections.

Among the new emerging strategies, the use of therapeutic monoclonal antibodies (mAbs) is gaining importance given their high affinity for the target, decreasing possible adverse effects.^29–31^ Until the present moment, many companies have sponsored clinical trials addressed to study hundreds of therapeutic mAbs for treating different diseases, from which 12 have been approved in EU (EMA) and/or US (FDA) by year 2024 to treat infectious diseases.^32,33^ The main targets of these mAbs are polysaccharides,^34^ surface proteins,^35^ bacterial virulence factors,^30^ and toxins.^36^ Sequestering nonessential cellular components instead of affecting microorganism viability constitutes an attractive therapeutic approach.

The synthesis of some of these targets is regulated by a bacterial process named quorum sensing (QS).^37^ This system enables bacterial communication through an increase in the levels of particular secreted small molecules called autoinducers (AIs). When bacterial density is high, the AI concentration reaches a threshold level that triggers changes in gene expression activating or inactivating important processes such as virulence, biofilm formation, and motility among others.^38^ Thus, disruption of QS mechanisms is being explored as an attractive alternative to fight against bacterial infections.^39–41^ It has been stated that blocking QS exerts less selective pressure than antibiotic treatment which should largely prevent the emergence of resistance mechanisms.^42–44^

In this regard, one of the main virulence factors of *P. aeruginosa*, pyocyanin (PYO), which is a phenazine regulated by the QS,^45^ shows potential as an interesting target. It has been reported that PYO exerts a large number of toxic effects on host cells^46^ mainly due to its redox properties and low molecular weight (210.23 g mol^–1^), which allows it to penetrate cytoplasmic membranes (cell permeability).^47^ This nitrogen-containing heterocyclic blue pigment can directly damage host tissues through the formation of short-lived reactive derivatives of oxygen species (ROS).^48^ The increase of intracellular ROS induces oxidative stress on the host, triggering free radical formation and damaging some cellular components, specially the mitochondria.^46^ Moreover, ROS exposure alters the production of cytokines,^49^ causes direct cellular damage and the interruption of cell signaling.^50^ In fact, it has been reported that PYO increases pro-inflammatory cytokines *in vitro* ^51^ and decreases anti-inflammatory cytokines *in vivo*.^52^ Focusing on the immune system, PYO also induces neutrophil apoptosis^53^ and can inhibit some macrophages, B and T cells’ function.^54–57^ Macrophages constitute the first line of defense in the airways^58^ being one of the main sources of cytokine production^59,60^ such as Tumor Necrosis Factor-α (TNF-α),^61^ interleukin-8 (IL-8),^62^ IL-6 ^63^ (pro-inflammatory cytokines), and also IL-10 and IL-13 ^64^ (anti-inflammatory ones).

Thus, this study aimed to assess PYO cytotoxic effects and to determine whether the specific antibody mAb122 can reduce this damage and the associated immune response dysregulation in murine macrophages. Specifically, it addressed the following question: Does mAb122 reduce PYO-induced cytotoxicity and immune dysregulation in host macrophages?

## 2 Results and Discussion

To initiate the study presented here, the development of a cell-based *in vitro* assay was a key requirement. For this purpose, two different macrophage cell lines were tested. Macrophages are relatively easy to cultivate, act as the first-line defenders against pathogenic bacteria, and initiate inflammation by releasing molecules such as cytokines. These three properties were relevant in the context of the work performed regarding PYO cytotoxicity and for the assessment of the mAb122 protective effect.

### 2.1 PYO Cytotoxic Effect

First, to optimize the assay length, cells were treated with different PYO concentrations during 24, 48, and 72 h and cytotoxicity was analyzed using AlamarBlue (AB) reagent. PYO concentrations tested were selected according to the levels reported in different biological samples such as ear secretions (up to 2.8 μM),^65^ sputum (up to 100 μM),^66^ and wounds (8.1 μM).^67^ The data obtained were fitted to a sigmoidal curve, which allowed characterization of the corresponding PYO lethal dose 50% (LD_50_) value. Based on the obtained results, MH-S and RAW 264.7 cells were grown for 72 h since it was the time required for PYO to exert its cytotoxic effect (data not shown) allowing to observe clear differences between treated and untreated cells. At day 3, PYO LD_50_ for MH-S cells (see Figure 1A) was slightly lower (15 μM) than that for RAW 264.7 cells (25 μM) (Figure 2). This difference might be due to the fact that the MH-S cell line was derived from lung tissue, one of the tissues most frequently infected by *P. aeruginosa*, specifically from the alveoli. Due to high susceptibility, the MH-S cell line was selected for more detailed cytotoxicity studies, analyzing other key viability hallmarks. Esterase activity, membrane integrity, and cell respiration were assessed using different viability assays. LD_50_ values ranged from 10 μM to 18 μM depending on the parameter studied (Figure 1B,C).

**Figure 1:**
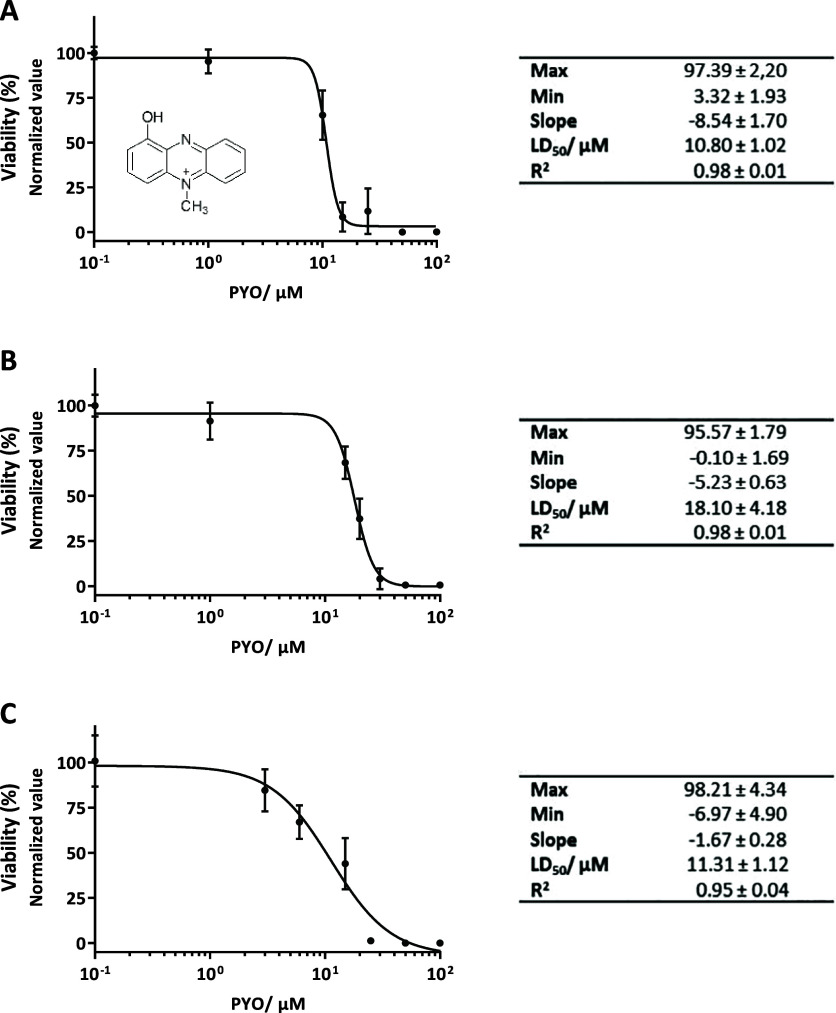
PYO cytotoxicity curves and parameters in MH-S cells. Cells were treated for 3 d with PYO concentrations ranging from 100 to 0 μM. The viability assays performed for these studies were (A) AlamarBlue (AB), (B) LIVE/DEAD, and (C) ATP Luminescence Kit. The results were measured in triplicates and the experiment was repeated minimum twice (data expressed as average and standard deviation). The obtained results were normalized according to the viability value of nontreated cells as it was considered 100% viability. The chemical structure of PYO is included in graph (A).

**Figure 2:**
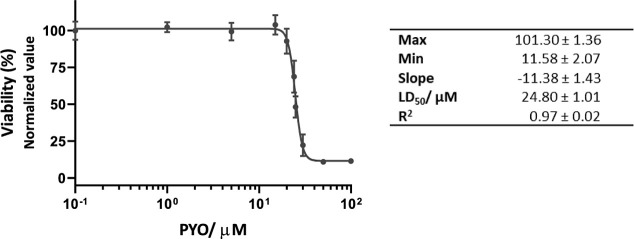
PYO cytotoxicity curve and parameters in RAW 264.7 cells. Cells were treated for 3 d with PYO at concentrations ranging from 100 to 0 μM. Cell viability was assessed using the AB assay and fluorescence values were measured at 530/590 nm. Experiments were performed in triplicate and repeated three times. Data are presented as mean ± standard deviation and normalized to the viability of untreated control cells (100%).

A comparison of the obtained results for each viability parameter on MH-S cells reveals that PYO exerted the greatest impact on mitochondrial enzymatic activity (AB) and cellular respiration (ATP assay), as indicated by the lower LD_50_ values determined in both cases. This effect is likely due to PYO redox activity, which increases ROS formation and inhibits cellular respiration. Moreover, the cytotoxicity data from both macrophage cell lines are consistent with previously reported results for this phenazine in other cell lines and assay conditions (see Table S1). Thus, Muller et al.^68^ treated human skin fibroblasts with different PYO concentrations (50 to 0 μM) for 24 h obtaining a LD_50_ value of around 10 μM when focusing on ATP production. In the same direction, O’Malley et al.^69^ also determined PYO LD_50_ on human alveolar type II cell line A549 looking at mitochondrial activity (MTT assay) and cell respiration (luciferase–luciferin assay). The LD_50_ values found in both cases were 30 μM. Furthermore, Moayedi et al.^57^ evaluated PYO cytotoxicity on a human pancreatic cancer cell line (Panc-1) using an XTT viability assay that as an AB reagent and MTT assays measure mitochondrial metabolic activity through a colorimetric reaction. After 24 h, the PYO LD_50_ on Panc-1 cell line was close to 45 μM. Finally, Mohammed et al.^70^ obtained a LD_50_ value of 32 μM when they treated hepatocellular carcinoma human (HepG2) cells with PYO for 72 h determining cellular viability with the neutral red dye assay, which is based on lysosomal activity.

### 2.2 PYO mAb122 Protective Effect

Using the same cell-based system, the protective value of a PYO specific mAb (mAb122) was analyzed by treating cells simultaneously with this antibody and PYO. The mAb effect was assessed in MH-S cells using the three viability markers previously studied in cytotoxicity assays. Additionally, mAb122 quenching was also evaluated in RAW 264.7 cells using only the AB reagent. Due to the long assay duration, we first checked PYO stability and mAb122 activity after 3 d of incubation under the conditions used to keep the cell culture in an optimal state (37 °C in an atmosphere with 5% CO_2_). For this, ELISAs were conducted using the supernatant of cells just treated with PYO (LD_50_) or just with mAb122 as an analyte or as a primary antibody. Besides, untreated cells and a PYO standard curve were also included as controls to ensure a correct viability assay performance. As shown in Figure S1, mAb122 kept its activity throughout the 3 d duration of the assay. This was demonstrated by the ELISA analytical parameters, which showed no significant differences between the standard curve generated using a freshly prepared antibody aliquot and that generated using the antibody recovered from cell culture supernatants (IC_50_ values of 4.75 and 5.22 nM, respectively). Comparable performance was also observed when cell supernatants from cultures treated solely with PYO were used as analytes in the ELISA (data not shown).

Following confirmation of immunoreagent stability, assays were conducted to evaluate the protective effect of the mAb122. MH-S untreated cells served as the 100% viability control, and all experimental results were normalized to this reference value. To assess the potential cytotoxic effects of the mAb itself, mainly caused by pyrogen contamination, a parallel control consisting of cells just treated with the mAb was included. Since PYO exerts most of its cytotoxic effects through ROS formation,^48,71^ mitochondrial enzymatic activity was considered an excellent cell viability hallmark to start performing these studies in both types of murine macrophages.

As illustrated in Figure 3A, the percentage of viable cells measured with AB increased 4.34-fold (from 10% to 43%, *p* < 0.0001) when a bivalent stoichiometric concentration of mAb122 was added to MH-S cells treated with 15 μM PYO (LD_50_), compared to cells just treated with the same PYO concentration. It should be noted that in these assays treatment with PYO at the LD_50_ caused more cell death than expected, which contributed to greater differences in viability percentages. In any case, the gap between one group and the other is clearly marked and statistically significant, further supported by the controls yielding the expected results.

**Figure 3:**
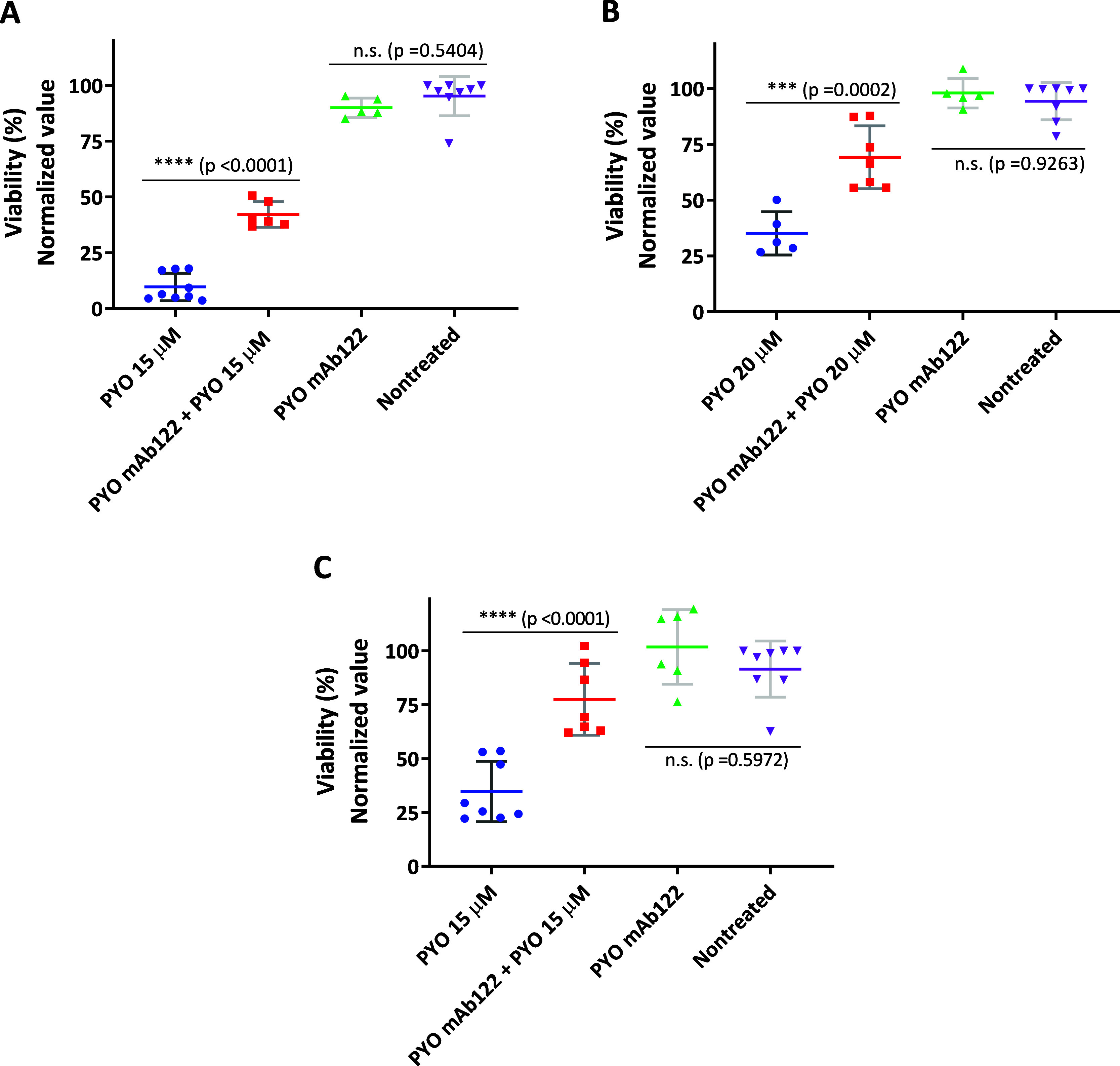
Protective effect of PYO mAb122 in MH-S cells. MH-S cells were treated with PYO (at the LD_50_ concentration) plus PYO mAb122 for 3 d. Subsequently, viability was estimated using (A) AlamarBlue (AB), (B) LIVE/DEAD, and (C) ATP kit viability assays. The viability of cells treated with PYO LD_50_ (blue) was compared with those of cells treated with the same PYO concentration plus a mAb stoichiometric concentration (red). Controls used include nontreated cells and cells treated just with the mAb. The obtained results were measured at least on duplicates and the experiment repeated at least 3 times. The results show the average and standard deviation of the values obtained. Green: wells with only a stoichiometric mAb concentration. Purple: nontreated cells. Data were normalized according to the viability value of nontreated cells as it was considered 100% viability.

Thus, the data obtained confirmed that mAb122 exerted a significant protective effect on the MH-S cells. Furthermore, it was corroborated that just mAb122 addition *per se* did not cause any harmful effect on MH-S cells as the percentage of viable cells remained similar (not significant) to the one obtained for nontreated cells.

This mAb122 quenching effect was also confirmed by studying membrane integrity and esterase activity (LIVE/DEAD dual staining). Figure 3B illustrates that the addition of mAb122 to MH-S cells treated with 20 μM PYO (corresponding to the LD_50_ for this viability parameter) resulted in a statistically significant (*p* = 0.0002) increase in cell viability, reaching approximately 70%. In contrast, treatment with PYO alone led to a viability of only 35%, indicating a 2-fold enhancement in cell survival due to the presence of mAb122. The results obtained also endorse the fact that mAb122 does not exert any cytotoxic effect on MH-S cells by itself.

Besides measuring fluorescence levels, this staining enabled us to obtain images showing the effects of both PYO and the corresponding antibody on MH-S macrophages. As illustrated in Figure 4, treatment with a PYO LD_50_ dose led to a decrease in cell viability, with only a small number of live cells remaining. No dead cells (red) were detected, likely due to the extended duration of the assay, which allows sufficient time for cells undergoing death to lose adhesion, often caused by cytoskeletal alterations. The protective effect of mAb122 is also clearly visible in the images, as cells treated simultaneously with the virulence factor and the quencher showed a marked increase in live (green) cells. The validity of the assay was confirmed using absolute ethanol as a negative control for cell viability, since it induces complete cell death. Additionally, macrophages were treated with the antibody alone to assess its potential cytotoxicity. As expected, a 30 min absolute ethanol treatment led to rapid and extensive cell death. Due to the short ethanol exposure time and its ability of cell fixation, the majority of dead cells remained attached to the plate. In contrast, treatment with the antibody alone once again confirmed its lack of cytotoxicity, as only a high number of viable (green) cells were observed.

**Figure 4:**
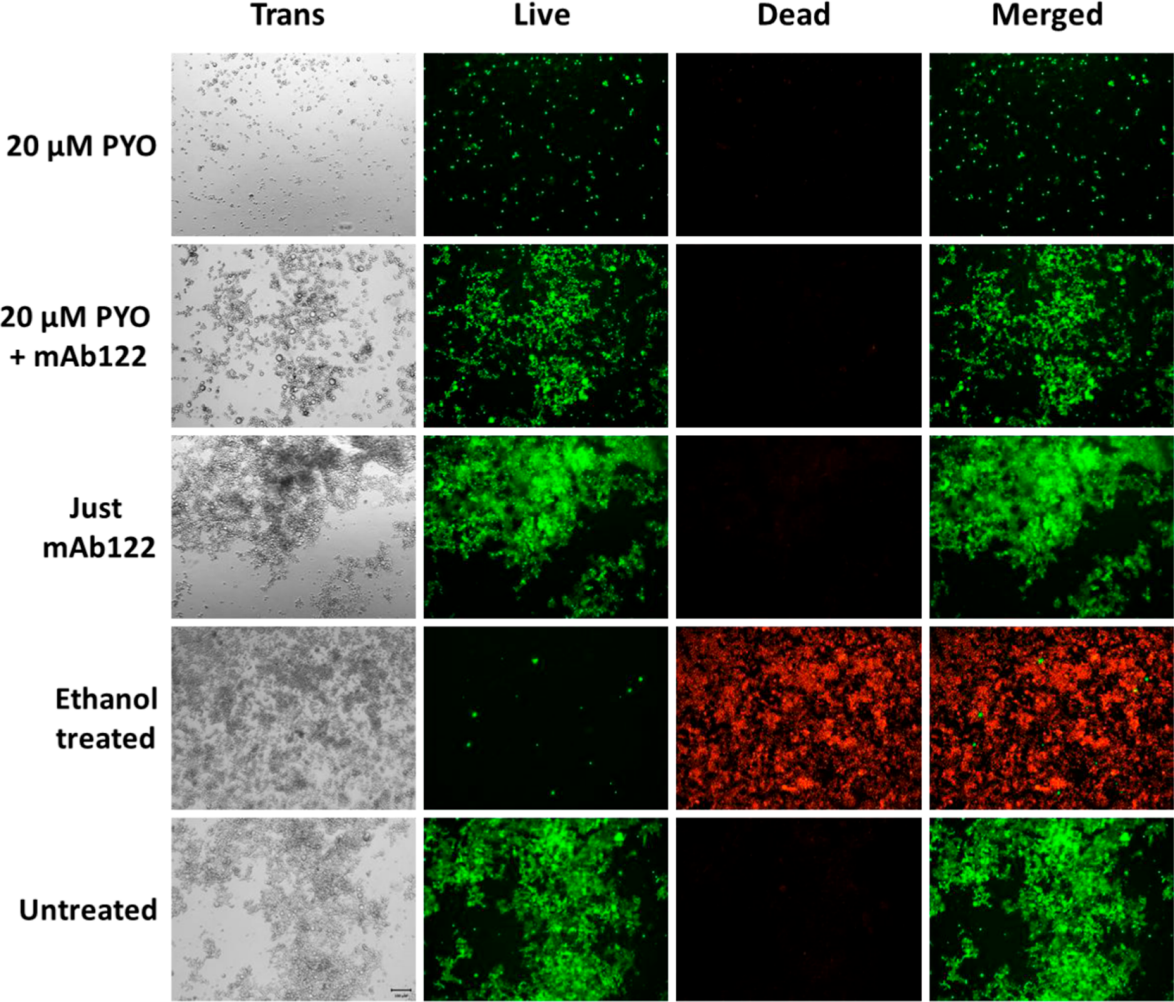
LIVE/DEAD fluorescent microscopy images of MH-S cells treated with PYO and with PYO and mAb122. MH-S cells were grown at 37 °C and 5% CO_2_ at a seeding density of 2 × 10^5^ cells mL^–1^ for 3 d. Calcein stains live cells (green fluorescence) and Propidium Iodide stains nucleic acids of dead cells (red fluorescence). As a negative viability control, cells were treated with absolute ethanol for 30 min, while untreated cells served as the positive control. Additionally, to assess any potential cytotoxic effect of the antibody itself, cells were treated with the quencher alone. Fluorescence labeling was monitored with an EVOS M7000 Imaging System at 10-fold magnification (scale bar: 100 μm).

Finally, Figure 3C demonstrates the protective effect of mAb122 on MH-S cells with regard to cellular respiration, a key viability parameter. Treatment with 15 μM PYO, corresponding to the LD_50_ for this assay, reduced the cell viability to 35%. However, co-treatment with mAb122 significantly improved viability, raising it to 78%, corresponding to a 2.2-fold increase. This enhancement of cell survival was statistically significant in all cases.

In order to ensure that the protective effect exerted by mAb122 was caused by its ability to specifically recognize and sequester PYO, the effect on the cell viability of an unrelated mAb was also studied. For this, a mAb specific for a different target^72^ such as streptomycin (STRC) was used as PYO quencher and its effect was evaluated by performing AB viability assays on MH-S cells. As shown in Figure S2, mAbSTRC did not provide any protection to MH-S cells. Cells treated with 15 μM PYO exhibited a viability of 33%, which was not significantly different from the viability of 29% observed in cells treated with both PYO and mAbSTRC, indicating a lack of protective activity. Importantly, mAbSTRC also showed no cytotoxicity toward MH-S cells, as viability in cells treated with the antibody alone (87.25%) was not significantly different from that of untreated controls (100%). Also to confirm the correct assay performance, mAb122 addition results were used as a control.

To strengthen our findings and confirm that the quenching effect of mAb122 is not limited to the selected cell line, we also included data demonstrating the protective activity of mAb122 in RAW 264.7 cells using AB (see Figure 5). To assess mAb122 protection against PYO-induced cytotoxicity, RAW 264.7 cells were treated with a PYO LD_50_ concentration (30 μM) in the presence or absence of the antibody. Co-treatment with a bivalent stoichiometric concentration of mAb122 significantly increased cell viability, yielding an 11.07-fold improvement, from 8.4% to 93.3% (*p* < 0.0001). Notably, the viability of mAb122-treated cells following PYO exposure was statistically indistinguishable from that of untreated controls (*p* = 0.6299), indicating complete neutralization of PYO’s cytotoxic effects.

**Figure 5:**
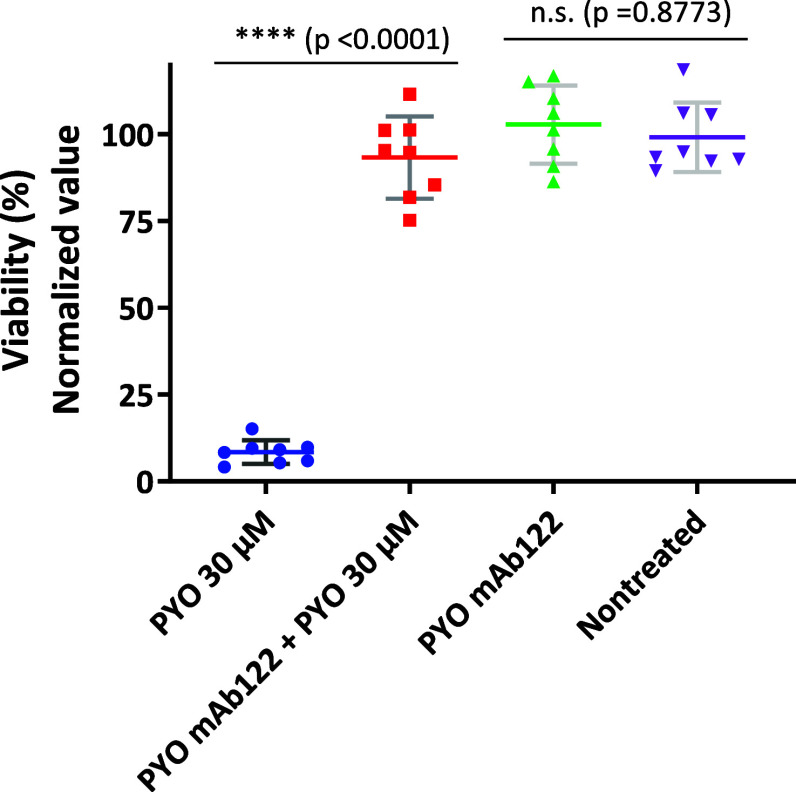
Protective effect of PYO mAb122 in RAW 264.7 cells. RAW 264.7 cells were treated with the LD_50_ value of PYO (30 μM) in the presence or absence of PYO mAb122 for 3 d. Viability was estimated also using AB reagent. The viability of cells treated with PYO at an LD_50_ concentration (blue) was compared with that of cells treated with the same PYO concentration plus a stoichiometric amount of mAb122 (red). Controls included nontreated cells and cells treated with mAb122 alone. Green: cells treated with mAb122 only. Purple: nontreated cells.

Furthermore, treatment with mAb122 alone had no impact on cell viability, as no significant difference was observed compared to untreated cells (*p* = 0.8773), confirming the antibody is noncytotoxic under the conditions tested. These results demonstrate the strong protective effect of mAb122 against PYO-induced cell damage, highlighting the specificity and efficacy of mAb122 in neutralizing PYO toxicity. Thus, protection was observed in two distinct macrophage cell lines with statistically significant results in both cases, strengthening the potential of the therapeutic strategy evaluated in this study.

In this sense, it should be emphasized that the use of antibodies as therapeutic agents has increased drastically in recent years, with over 115 antibodies currently (year 2022) approved by the FDA and EMA.^59^ The first therapeutic mAb (Orthoclone OKT3) was approved by the US FDA in 1986 to prevent kidney transplant rejection^73–75^ and since then the cancer treatment dominates the antibody market (42.6% of FDA approved antibodies). However, mAbs are increasingly used in other clinical areas such as immune-related diseases, hematology, neurology, and infectious diseases.^75^ Regarding these last ones, currently, 12 mAbs are approved in the EU and/or US for infectious diseases, targeting diseases such as inhalational anthrax, *Clostridium difficile* infection, respiratory syncytial virus (RSV), HIV, Ebola virus disease, and COVID-19. The use of these therapeutic mAbs has proven as a promising alternative to treat these types of diseases since resistance can be diminished and even avoided due to their high specificity. Nevertheless, some authors advocate for using this approach in combination with low doses of antibiotics rather than as a single therapy.^76^ Moreover, in the field of infectious diseases, antivirulence therapies are gaining ground as a compelling alternative to conventional antimicrobial compounds, especially due to the serious issue of the development of AMR processes. Unlike traditional antibiotics, antivirulence therapies do not aim at eradicating bacteria but to disarm and make them less harmful, allowing the host immune system to clear the infection easily and exerting less selective pressure for resistance. Examples of antivirulence strategies include mainly: toxin neutralizers, secretion systems, and QS inhibitors.

Antitoxins, for instance, are already applied in diphtheria therapy.^77^ Other strategies focus on blocking bacterial secretion systems, with several inhibitors of the Type III Secretion System (T3SS), such as mAbs^78–80^ and small molecules,^81^ showing protective effects in experimental and early clinical trials,^82^ although their development faces limitations.

Finally, blocking or attenuating bacterial communication (quorum quenching, QQ) is a very attractive approach for developing new therapeutic strategies. Quenching key targets of QS pathways not only disrupts bacterial communication process but also blocks pathogenic outcomes,^83^ including toxic or immunomodulatory effects caused by QS molecules and QS-regulated virulence factors.^46^ Disruption of the QS system has mainly focused on three strategies acting at different steps of QS circuits:^84–86^ First, interfering with the generation of the inducing signal (AI) by inhibiting synthases or efflux pumps.^87^ Data on this are limited and mainly concerns autoinducer 2 (AI-2)^88^ and homoserine lactones (HSLs).^89^ Although not categorized as virulence factors, some AIs exert cytotoxic effects on host cells,^90–92^ and their inhibition reduces virulence factor production and biofilm formation.^93^ Second, receptor binding can be blocked with AI analogs. Numerous studies report natural bioactive molecules and synthetic products acting as QQ agents.^87,94,95^ Third, QS molecules can be degraded or modified by enzymes such as lactonases, acylases, and oxidoreductases.^96^ In this sense, the use of molecules that act as scavengers or sequesters, such as antibodies or antibody fragments, has also been reported,^97^ and it is the basis of the case reported here. One example is Janda’s mAb RS2-1G9 against *N*-(3-oxododecanoyl)-l-homoserine lactone (3-oxo-C12-HSL), which protected murine bone marrow-derived macrophages from PYO cytotoxicity and prevented activation of the mitogen-activated protein kinase p38, a biochemical marker of cytotoxicity.^98,99^ Despite these promising findings, there is no evidence that RS2-1G9 has advanced to clinical trials or has received regulatory approval.

#### 2.2.1 PYO Immunomodulatory Effect

Antibody-based therapy directed to QS regulated targets should not only block bacterial virulence but also minimize the host immunity dysregulation that is being modulated/suppressed by the QS system. It has been reported that PYO induces an inflammatory response on airway epithelial cells^100^ by increasing pro-inflammatory cytokines *in vitro* ^49,101^ and decreasing anti-inflammatory cytokines *in vivo*.^52^ In this context, we aimed to test whether adding a quencher to PYO-treated macrophages could prevent the dysregulation of the host immune response caused by this virulence factor by also minimizing the PYO cytotoxic effect. To do this, we first determined the effect of PYO treatment on the cytokine levels produced by our main cell model (MH-S cells) and also analyzed the basal levels of the selected cytokines in untreated cells. The cytokines measured using commercial ELISAs were chosen according to the already published literature focused on PYO modulation of the host immune response in *in vitro* conditions^49,100,101^ in different cell lines. Thus, IL-8, IL-6, and TNF-α pro-inflammatory cytokines were quantified in macrophage supernatants since these types of cells are one of the main producers of these molecules.^58,60,63^ Moreover, IL-13 anti-inflammatory cytokine was also analyzed as it has been demonstrated that PYO decreases basal IL-13 levels in mice.^51^ To carry out these assays, MH-S cells were treated with a PYO concentration equivalent to the corresponding LD_50_ value and two concentrations below this one were also assayed (15, 6, and 3 μM).

As a general trend, the results demonstrated a PYO dose-dependent increase in pro-inflammatory cytokine levels, with a more pronounced effect observed for IL-6 and IL-8 compared to TNF-α (see Figure 6A–C). In all cases, treatment with a PYO LD_50_ concentration led to a statistically significant increase in cytokine levels relative to those measured in supernatants from untreated cells. In contrast, PYO had no apparent immunomodulatory effect on IL-13, as none of the tested concentrations induced statistically significant changes in the levels of this anti-inflammatory cytokine (Figure 6D).

**Figure 6:**
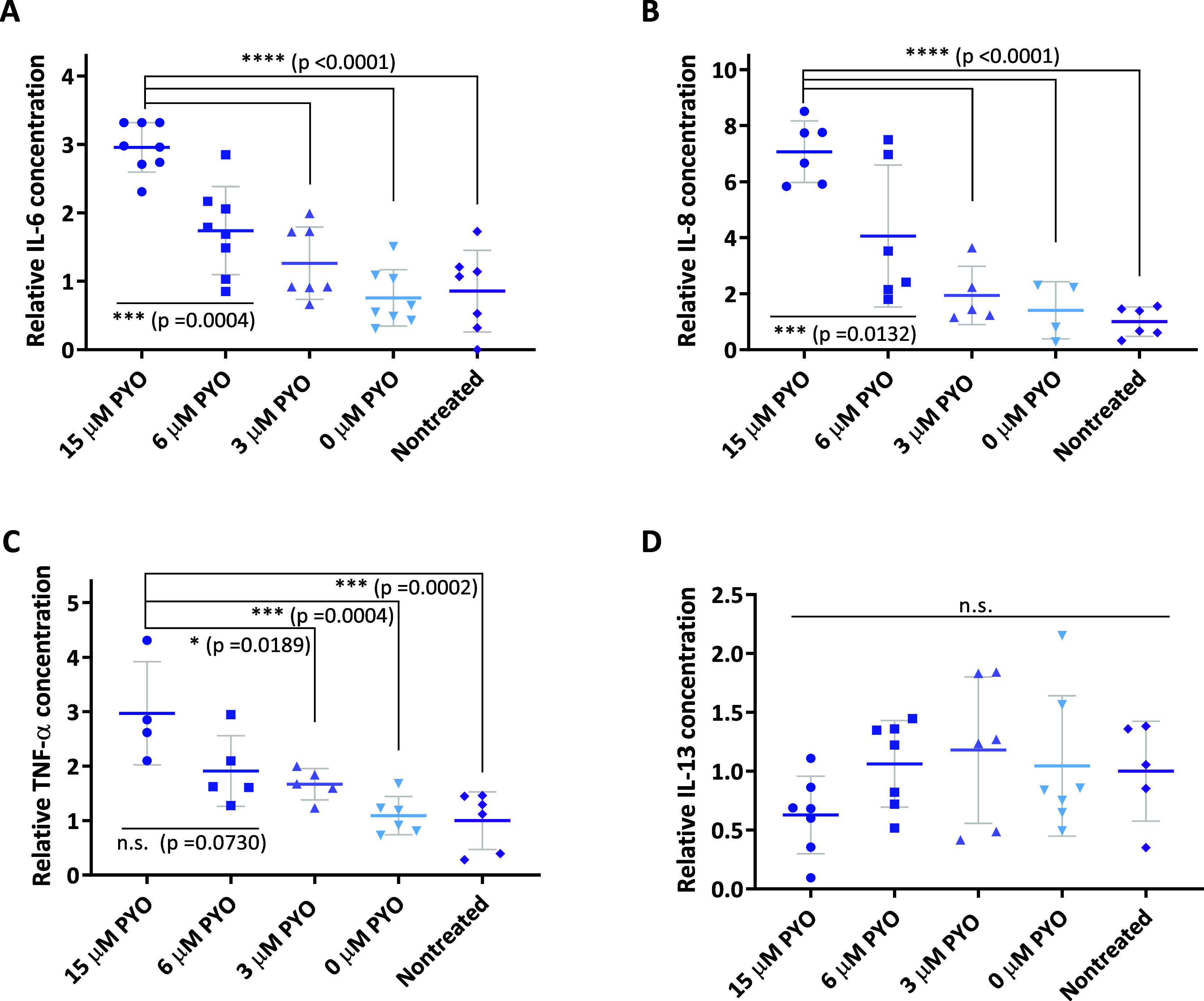
(A) IL-6, (B) IL-8, (C) TNF-α, and (D) IL-13 levels measured in media of MH-S cells treated with different PYO concentrations during 3 d. Measures were performed by ELISA. 15 μM corresponds to the PYO LD_50_ on MH-S cells determined using AlamarBlue (AB). The obtained results were measured at least on duplicates and the assay repeated minimum twice. The results show the average and standard deviation of the data obtained. Values were normalized according to the maximum value recorded in each assay.

Reported *in vitro* studies have identified that PYO induces IL-8 expression in a dose-dependent manner producing a measurable IL-8 increase at 5 μM PYO concentration after 24 h exposure on human airway epithelial cells.^101^ Similarly, Chai et al.^102^ demonstrated that PYO stimulates IL-8 expression in U937 monocyte cells as a function of the virulence factor concentration and time of exposure. Thus, cells treated with 25 μM PYO increased 3 times the secretion of this potent neutrophil chemoattractant just after 2 h of treatment. Other pro-inflammatory cytokines, such as IL-6 and TNF-α, are also upregulated due to PYO presence as confirmed by Rada et al.^49^ In fact, PYO exposure (8 μM) in tracheobroncheolar epithelial cells (TBECs) translated into a 7-fold and 4-fold increase in IL-6 and TNF-α transcriptional levels after 48 h of exposure quantified using a human whole genome microarray. Furthermore, this increase was also analyzed by real-time PCR, finding that IL-6 and TNF-α levels showed even a more marked increase of 391- and 26-fold times, respectively. Regarding anti-inflammatory cytokines, Larian et al.^52^ demonstrated that PYO treatment decreased IL-13 levels on mice. In this case, mice were administered PYO escalating doses (2, 6, 19, and 50 mg kg^–1^) by intraperitoneal injection and 24 h after the last PYO boost IL-13 levels were determined in plasma resulting into a significant decrease (*p* < 0.05). In agreement with all these previously reported data, PYO treatment on MH-S cells also produced a dose-dependent increase of pro-inflammatory cytokine levels after 72 h of exposure. This immunomodulatory effect was only significant when the PYO LD_50_ concentration was tested and just for IL-6, a lower PYO concentration (6 μM) also caused a statistically relevant increment of this cytokine levels (Figure 6A–C). On the contrary, IL-13 levels were not affected by PYO treatment (Figure 6D), which could be attributed to the cell line used as a model or to the experimental conditions set up. In any case, PYO main effect consists of generating oxidative stress, triggering pro-inflammatory responses rather than anti-inflammatory ones.

Subsequently, mAb122 was added to treated cells to sequester PYO and reduce its immunomodulatory effect on MH-S cells. Figure 7A–D shows that, in contrast to the expected outcome, treatment with mAb122 did not attenuate PYO immunomodulatory effects on MH-S cells. Specifically, the addition of mAb122 failed to reduce PYO-induced increase on IL-6 and IL-8 levels (Figure 7A,B). Moreover, treatment with just mAb122 resulted in a statistically significant increase in both cytokines compared to untreated cells, showing levels comparable to those observed following PYO exposure. Regarding TNF-α, its levels were the least affected by PYO treatment, but whenever mAb was added, it caused a significant rise in this immunomodulatory molecule concentration with respect to cells treated with PYO and to nontreated cells (Figure 7C).

**Figure 7:**
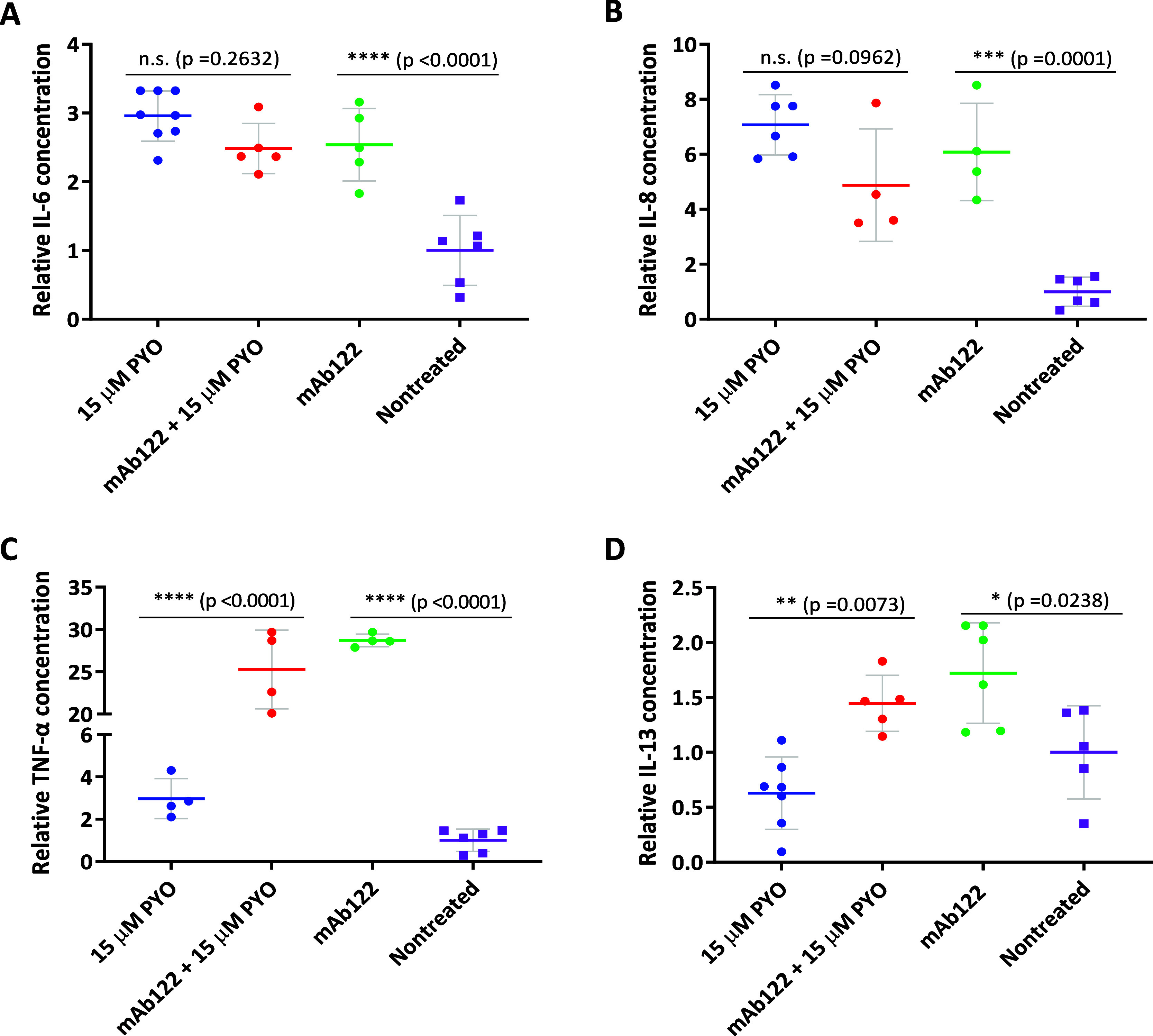
(A) IL-6, (B) IL-8, (C) TNF-α, and (D) IL-13 levels in media of MH-S cells treated (blue) with 15 μM PYO, (red) 15 μM PYO plus PYO mAb122, (green) only PYO mAb122, and (purple) nontreated cells for 3 d, using the corresponding ELISA. The obtained results were measured at least on duplicates and the assay repeated at least twice. The results show the average and standard deviation of the data obtained. Values were normalized according to the maximum value obtained in each assay.

Notably, IL-13 levels exhibited a pattern similar to that of the pro-inflammatory cytokines studied (see Figure 7D). Treatment with mAb122, in both the presence and absence of PYO, resulted in a significant increase in IL-13 levels compared to cells treated with PYO alone and to untreated controls. Thus, the results from MH-S cells showed that mAb122 did not minimize PYO-induced immune dysregulation. On the contrary, the antibody alone significantly increased cytokine levels compared to untreated cells, and in some cases, it triggered higher levels than cells treated just with PYO. This increase could be attributed to a frequent inflammatory response of the cellular immune system called the cytokine-release syndrome (CRS).^103^

In fact, CRS is considered a common immune-mediated toxicity effect in which macrophages and monocytes produce different cytokines, with IL-6 being the central mediator of toxicity.^104,105^ This syndrome is usually observed as a response to an infection or immunotherapy treatment. In this sense, it has been reported that *in vivo* infusion of mAbs, such as daclizumab and rituximab, activated this syndrome within the first 2 to 48 h of treatment.^106^ CRS is usually expected to be mild, have prognosis, and resolve over time.^106^ CRS has been evaluated in various cell lines (human peripheral blood mononuclear cells (PBMCs) and whole blood assays), however, these systems do not exactly predict the cytokine release that occur in *in vivo* experiments nor in patients.^107^ Therefore, to confirm the potential induction of CRS, it will be essential to evaluate mAb122 in an *in vivo* model to determine whether its administration elicits adverse effects. Under these conditions, identifying the most appropriate mAb delivery system will also be critical. Furthermore, in the present case, alternative strategies could be considered to mitigate or even prevent the onset of this syndrome. First, other PYO mAb clones can be assayed, and even other mAbs could be tested against other target molecules of the QS system. Furthermore, the use of antibody fragments or nanobodies (Nbs) could also be considered, as their small size enhances the pharmacokinetic and pharmacodynamic profiles of these quenchers.

The cell viability protection effect of mAb122 provides a strong foundation for advancing preclinical studies, including both *in vitro* and *in vivo* assays, which will also need to account for the host immune response to antibody treatment. These findings may also support future exploration of the clinical therapeutic potential of mAbs targeting other virulence factors regulated by QS or even mAbs specific to key components of the QS system itself. Furthermore, this opens new avenues for investigating alternative antibody formats such as Nbs or QS-bispecific antibodies as potential quenchers. In addition, advances in cell culture techniques that better mimic tissue-level functionalities are expected to greatly aid this field by enabling more accurate studies of host–pathogen interactions and facilitating the validation of antibody-based quenchers, ultimately allowing progression to *in vivo* trials in a safer and more reliable manner.

In the context of antimicrobial resistance, this therapeutic approach offers a significant advantage, as it does not depend on directly killing the pathogen. Several comprehensive reviews highlight the numerous benefits of QS-related antivirulence strategies, which appear to outweigh their potential limitations.^44,108^ Furthermore, some authors propose that the most effective strategy for treating infectious diseases may involve combining antivirulence quenchers with low doses of antibiotics, thereby contributing to reduce antibiotic overuse.^76,109^ This study provides compelling evidence of the therapeutic potential of a mAb-targeting PYO, a key virulence factor regulated by the QS system, under *in vitro* conditions. Notably, this antibody has been shown to play a crucial protective role in preserving the viability of macrophages exposed to this phenazine. These promising findings pave the way for *in vivo* studies to validate the protective effect of antibodies as therapeutic agents and to unravel the host immune response to this treatment, confirming the clinical relevance of this innovative approach.

## 3 Materials and Methods

### 3.1 Cell Line Models

MH-S cells (CRL-2019, ATCC) (kindly given by Dr. A Lacoma (HUGTP, Badalona, Spain)), a murine alveolar macrophage adherent cell line, were cultured in Roswell Park Memorial Institute (RPMI 1640) medium (Gibco) supplemented with 10% heat inactivated fetal bovine serum (FBS, Sigma), 10 mM HEPES (Gibco), and 1% Antibiotic/Antimycotic 100× (Gibco). Cells were maintained at 37 °C and 5% CO_2_ in a humidified atmosphere.

RAW 264.7 cells (ATCC TIB-71), a murine cell line from Abelson leukemia virus-induced tumor, were grown in Dulbecco’s Modified Eagle’s Medium (DMEM, Sigma-Aldrich Co, St. Louis) supplemented with 10% heat inactivated FBS, 1% nonessential amino acids (Sigma), and 1% antibiotic/antimycotic 100× (Gibco). Cells were incubated in a humidified atmosphere at 37 °C and 5% CO_2_.

### 3.2 PYO mAb and ELISA

The PYO mAb (clone 122) used in the experiments presented in this paper was generated in-house by the Nb4D group (IQAC–CSIC), with the support of the Custom Antibody Service (CAbS, IQAC–CSIC, CIBER-BBN). Its production and characterization were published by Rodriguez-Urretavizcaya et al.^110^ MAb122 is a high-affinity antibody raised against the PYO precursor 1-hydroxyphenazine (1-OHphz). Through selective screening using both PYO and 1-OHphz, and after several cloning cycles, a clone capable of detecting PYO at low concentrations was selected. Using this mAb122, a highly sensitive, specific, and reliable immunochemical assay (ELISA) was developed to detect PYO, achieving a limit of detection (LoD) of 0.07 nM. It is also important to note that the antibody also recognizes 1-OHphz with high detectability. However, in the context of the experiments conducted in this study, this cross-reactivity is not relevant, although it will be considered in future preclinical studies.

The protocol followed to carry out PYO ELISA was the one described in Rodriguez-Urretavizcaya et al.^110^

### 3.3 *In Vitro* Assay Protocols

#### 3.3.1 Cytotoxicity Assays

Cells were plated in 96-well plates (different types of plates were used for each sort of viability assay performed) at a seeding density of 2 × 10^5^ cells well^–1^ in a final volume of 100 μL and incubated for 2 h in complete medium for cell adhesion. Then, medium was changed to remove nonadhered cells and the seeded ones were further treated with different PYO (Sigma-Aldrich Co, St. Louis) concentrations and incubated for 3 d in the corresponding medium. PYO concentrated solution was prepared in DMSO (Dimethyl sulfoxide) (Sigma-Aldrich Co, St. Louis) and subsequent dilutions (concentrations ranging from 100 to 0 μM, prepared with different dilution factors depending on the viability hallmark studied) were made in the appropriate medium depending on the macrophage line (without exceeding 1% DMSO, the limit tolerated by the cells). Cell viability percentage and LD_50_ values were estimated according to assay manufacturer instructions.

#### 3.3.2 mAb Protective Effect Assay

The protective effect of mAb122 on cultured MH-S cells was evaluated using a protocol similar to that described above. Cells were seeded in 96-well plates, as previously detailed in the preceding section. In each plate, some wells were treated with the previously determined LD_50_ PYO concentration alone, while others were coincubated with mAb122 and PYO LD_50_ value. The Ab concentration used was calculated based on the PYO LD_50_ value to finally add a stoichiometric amount of it considering that each mAb molecule binds two PYO molecules. Moreover, in each assay, a PYO standard curve was also built to ensure the correct performance of the assay. Viability percentages of cells just treated with PYO were compared with those of cells treated with the quencher (mAb122) plus the virulence factor. As for cytotoxicity assays, plates were incubated for 3 d at 37 °C and 5% CO_2_. After this period of time, commercial viability assays manufactured to study different viable cell characteristic processes were carried out to evaluate the mAb protective effect.

#### 3.3.3 Viability Assays

To address PYO cytotoxicity and the mAb protective effect, different commercial kits were used to study 3 different specific hallmarks of viable cells: mitochondrial enzymatic activity, esterase activity and membrane integrity, and cellular respiration. With this aim, the AlamarBlue (AB) reagent (Invitrogen, DAL1025), LIVE/DEAD dual staining (Invitrogen, L3224) and Luminescent ATP Detection Assay kits (Abcam, ab113849) were purchased.

### 3.4 Mitochondrial Enzymatic Activity

The AlamarBlue (AB) Cell Viability Reagent (Invitrogen, DAL1025) allows the detection of viable cells taking advantage of the cellular metabolic function itself.^111^ Thus, AB is a resazurin (7-hydroxy-3*H*-phenoxazin-3-one 10-oxide)-based solution that works by using the reducing power of living cells to quantitatively measure viability. Reduction of resazurin takes place intracellularly and involves mitochondrial reductases and other enzymes producing a pink and fluorescent compound called resorufin.^112,113^ The quantity of the produced resorufin is proportional to the number of viable cells and is quantified by fluorescence at an excitation/emission wavelength of 530/590 nm.

The assay was carried out in Nunclon Delta-treated 96-well flat bottom microwell plates (Thermo Fisher Scientific, 167008). After 3 d of incubation, medium was removed, cells were washed with 10 mM phosphate-buffered saline (PBS) (100 μL well^–1^), and a solution of AB reagent 12 times diluted in medium (100 μL well^–1^) was added to each well. Then, the plate was incubated for 4 h at 37 °C shaking at 600 rpm. Finally, cell supernatants were diluted 20 times in 10 mM PBS in a Nunc F96 microwell white polystyrene plate (Thermo Fisher Scientific, 236107) (200 μL well^–1^) for fluorescence (*F*) measurements performed in a SPECTRAmax GEMINI XS spectrofluorometer. Results were expressed as

1 $$\%\;viability=\frac{F\;experimental\;cell\;sample}{F\;non\;treated\;cells}\times 100$$

### 3.5 Cell Membrane Integrity and Esterase Activity

Esterase activity and plasma membrane integrity hallmarks of viability were analyzed using the LIVE/DEAD Viability/Cytotoxicity Kit (Invitrogen, L3224). This assay relies on the uptake of the nonfluorescent cell-permeant dye calcein acetoxymethyl (CA-AM) by living cells and the staining of dead cells by the incorporation of the fluorescent DNA intercalator ethidium homodimer-1 (EthD-1). CA-AM is hydrolyzed by intracellular esterases to CA, which produces an intense fluorescent green signal at 530 nm. Besides, EthD-1 penetrates into cells with damaged membranes, allowing its binding to the nucleic acids existing in the nucleus, resulting in a fluorescent red signal at 645 nm.

The assay was performed in 96-well Nunclon Delta treated black/clear bottom microwell plates (Thermo Fisher Scientific, 165305). In this case, cells were seeded at 2 × 10^5^ cells mL^–1^ and after the corresponding treatment for 3 d, cells were washed with 10 mM PBS (100 μL well^–1^) prior to the addition of a solution containing 4 μM CA-AM and 4 μM EthD-1 diluted in 10 mM PBS (100 μL well^–1^). Cells were then incubated for 1 h at room temperature (RT) and finally fluorescence was measured at 485/530 nm (for CA) and 530/645 nm (for EthD-1). Results were expressed as

2 $$\%\;viability=\frac{F\;530\;nm\;of\;cell\;sample\;labeled\;with\;CA‐AM\;\text{and}\;EthD‐1-F\;530\;nm\;where\;\text{all}\;cells\;alive\;labeled\;with\;EthD‐1}{F\;530\;nm\;where\;\text{all}\;cells\;alive\;labeled\;with\;CA‐AM\;\text{and}\;EthD‐1-F\;530\;nm\;where\;\text{all}\;cells\;alive\;labeled\;with\;EthD‐1}\times 100$$

Fluorescent images were acquired with an EVOS M7000 Imaging system (Thermo Fisher), acquiring them at 10× magnification and keeping the exposure settings constant for all samples. In this case, as a negative control, cells were treated with absolute ethanol (100 μL well^–1^; Panreac ref. 251085) for 30 min prior to dye addition to induce cell death.

### 3.6 Cellular Respiration

The third studied viability trait was the adenosine triphosphate (ATP) levels. The ATP molecule is synthesized by viable cells through aerobic respiration to obtain energy. Thus, it is an adequate indicator of metabolically active cells.^114^ The Luminescent ATP Assay Kit (Abcam, ab113849) was used to quantify the ATP levels produced by MH-S cells. The principle behind this methodology relies on luciferase’s requirement for ATP to be able to produce light. The assay was conducted according to manufacturer instructions in the same 96-well plates used for the AB assay (Thermo Fisher Scientific, 167008). Briefly, after 3 d PYO treatment, cell lysis was induced adding a detergent solution that contains ATPase inhibitors (50 μL well^–1^) for 5 min at RT and shaking at 600 rpm. Subsequently, the luciferase stable form and luciferin substrate were added (50 μL well^–1^) and incubated as in the step before.^115^ Finally, the plate was placed in the dark for 10 min and 100 μL of the corresponding cell lysate samples was transferred into 96-well white microwell plates (Thermo Fisher Scientific, 236107) for bioluminescence lecture at 570 nm (Glomax Multi Detection System, Promega). A standard curve with ATP standard solutions was run in each experiment. Results were expressed as

3 $$\%\;viability=\frac{bioluminiscence\;of\;cell\;sample}{bioluminiscence\;of\;non\;treated\;cells}\times 100$$

### 3.7 PYO Immunomodulatory Effect Studies

The immunomodulatory effect of PYO on MH-S cells was evaluated following the same protocol described in Section 2.2.1. In this experiment, cells were treated with different PYO concentrations (15, 6, 3, and 0 μM), with mAb122 alone, or with both PYO and mAb122. After 3 d of incubation at 37 °C in a humidified atmosphere with 5% CO_2_, cell culture supernatants were collected for cytokine quantification. IL-6, IL-8, TNF-α, and IL-13 levels were measured using sandwich ELISAs. Supernatants were stored at −20 °C until analysis.

### 3.8 Cytokine Immunoassays

The selected cytokines (IL-6, IL-8, TNF-α, and IL-13) were quantified by ELISA using commercially available kits (Sino Biological). Briefly, microtiter 96-well ELISA plates were coated with the corresponding capture mAb diluted in 10 mM PBS (100 μL well^–1^) and incubated overnight (ON) at 4 °C. Next day, the plates were washed 3 times with 0.05% Tween 20 in 10 mM PBS (PBST) and blocked with 1% bovine serum albumin (BSA) in 10 mM PBS (100 μL well^–1^) for 30 min shaking at 600 rpm and kept at RT. After the blocking step, cell supernatants and cytokine standards were added (50 μL well^–1^) and incubated for 2 h at RT shaking at 600 rpm. Cytokine standards were prepared in RPMI media given the matrix effect results observed (data not shown). Subsequently, the corresponding detection mAb labeled with peroxidase was added in 10 mM PBST (100 μL well^–1^) and left for 1 h at RT shaking at 600 rpm. Finally, Ab binding was quantified by adding the substrate solution (3,3′,5,5′-tetramethylbenzidine (TMB) and H_2_O_2_ solution) (100 μL well^–1^). This reaction was stopped with 4 N H_2_SO_4_ (50 μL well^–1^) and the absorbance was then measured at 450 nm.

### 3.9 Statistical Analysis

The results are expressed as mean ± standard deviation (SD) and analyzed with GraphPad Prism 7.0. software (GraphPad Software, Inc., San Diego, CA) using one-way variance analysis (ANOVA) and Tukey’s multiple comparison test. For viability assays, wells containing untreated cells were considered as 100% viability. All cytotoxicity and mAb protective effect results were normalized according to that value. Cytokine levels were normalized according to the higher value obtained for each assay.

## Supplementary Material



## Notes

Stability studies of PYO mAb122 using PYO mAb122/PC1-BSA ELISA, specificity studies of the PYO mAb122 effect in MH-S cells, and reported LD_50_ values for PYO on different cell lines (PDF)

## Abbreviations

3-oxo-C12-HSL: *N*-(3-oxododecanoyl)-l-homoserine lactone
AB: alamarblue reagent
AI: autoinducer
AMR: antimicrobial resistance
ATP: adenosine triphosphate
BPPL: bacterial priority pathogens list
COPD: chronic obstructive pulmonary disease
CRS: cytokine-release syndrome
ELISA: enzyme-linked immunosorbent assay
EMA: European Medicines Agency
FDA: Food and Drug Administration
HAI: hospital-acquired/associated infection
HSL: homoserine lactone
IL-6, 8, 13: interleukin 6, 8, 13
LD_50_: lethal dose 50%
LTRI: lower tract respiratory infection
mAb: monoclonal antibody
mAbSTRC: monoclonal antibody specific for streptomycin
Nb: nanobody
PYO: pyocyanin
QS: quorum sensing
ROS: reactive oxygen species
T3SS: type III secretion system
TNF-α: tumor necrosis factor α
WHO: World Health Organization
