# Supplementary material for: Anti-pyocyanin Antibody Exhibits Cytotoxicity Protective Effects on Macrophages: A Promising Innovative Therapeutic Approach for Pseudomonas aeruginosa Infections
Source: ACS Pharmacol Transl Sci. 2025 Nov 6;8(12):4248–63. doi: 10.1021/acsptsci.5c00187 (PMC12706814; doi:10.1021/acsptsci.5c00187)
Supplement: Supplementary file 1 [file pt5c00187_si_001.pdf]

Suppporting Information

Anti-Pyocyanin Antibody exhibits Cytotoxicity Protective Effects on Macrophages: A Promising Innovative Therapeutic Approach for *Pseudomonas aeruginosa* Infections

Bárbara Rodríguez-Urretavizcaya[1,2], Tamás Posvai[1], Lluïsa Vilaplana[1,2*] and María-Pilar Marco[1,2]

[1]Nanobiotechnology for Diagnostics (Nb4D). Institute of Advanced Chemistry of Catalonia, IQAC-CSIC. Jordi Girona, 18-26, 08034-Barcelona, Spain.

[2]CIBER de Bioingeniería, Biomateriales y Nanomedicina (CIBER-BBN). Av. Monforte de Lemos, 3-5. Pabellón 11. Planta 0 28029 Madrid

[*]To whom correspondence should be sent:

Phone: + 34 93 4006100. Fax: + 34 93 2045904.

e-mail: lluisa.vilaplana@iqac.csic.es

Table of Contents

Figure S1. *Stability studies of PYO mAb122 using PYO mAb122/PC1-BSA ELISA.* 3

Figure S2. *Specificity studies of PYO mAb122 effect in MH-S cells.* 4

Table S1. *Reported LD_{50} values for PYO on different cell lines.* 5

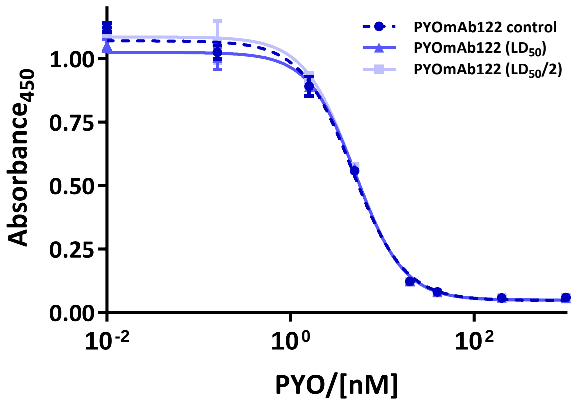

Figure S1. *Stability studies of PYO mAb122 using PYO mAb122/PC1-BSA ELISA. PYO mAb122 was added to MH-S cells at the LD_{50} concentration (middle blue) and half of the LD_{50} concentration value (light blue) and incubated for 3 d at 37 ºC and 5 % CO_2. Cell media was used as primary Ab to perform PYO mAb122/PC1-BSA ELISA according Rodriguez-Urretavizcaya et al[65](standard reagents and conditions in dark blue).*

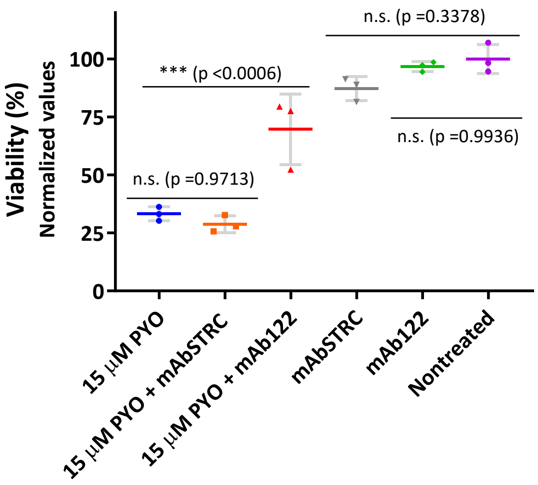

Figure S2. *Specificity studies of PYO mAb122 effect in MH-S cells. MH-S cells were treated with (blue) 15 µM PYO, (orange) 15 µM PYO plus mAbSTRC, (red) 15 µM PYO plus PYO mAb122, (grey) mAbSTRC (green) PYO mAb122 and (purple) non treated MH-S cells for 3 days. After that time, AlamarBlue (AB) viability assay was performed. The obtained results were measured on triplicates and the results show the average and standard deviation. The obtained results were normalized according to the viability value of non-treated cells as it was considered the 100 % of viability.*

Table S1. *Reported LD_{50} values for PYO on different cell lines.*

Cell line	LD_{50}	Cytotoxicity assay	Reference
Human skin fibroblasts	10 µM	CellTiter-Glo luminescent Cell Viability Assay kit	Muller et al.[108]
Human pancreatic cancer cell line (Panc-1)	44.84 µM	XTT assay	Moayedi et al.[57]
Hepatocellular carcinoma human (HepG2)	31.5 µM	Neutral red dye	Mohammed et al.[110]
The human alveolar type II cell line A549	30 µM	MTT assay	O'Malley et al.[109]

MTT =3-(4,5-dimethylthiazol-2-yl)-2,5-diphenyltetrazolium bromide; XTT =2,3-Bis-(2-Methoxy-4-Nitro-5-Sulfophenyl)-2H-Tetrazolium-5-Carboxanilide
